# Antimicrobial and Antibiofilm Activity of Synergistic Combinations of a Commercially Available Small Compound Library With Colistin Against *Pseudomonas aeruginosa*

**DOI:** 10.3389/fmicb.2018.02541

**Published:** 2018-10-25

**Authors:** Nelson S. Torres, Daniel Montelongo-Jauregui, Johnathan J. Abercrombie, Anand Srinivasan, Jose L. Lopez-Ribot, Anand K. Ramasubramanian, Kai P. Leung

**Affiliations:** ^1^Dental and Craniofacial Trauma Research and Tissue Regeneration Directorate, Institute of Surgical Research, San Antonio, TX, United States; ^2^Department of Biomedical Engineering, The University of Texas at San Antonio, San Antonio, TX, United States; ^3^Department of Biology, South Texas Center for Emerging Infectious Diseases, The University of Texas at San Antonio, San Antonio, TX, United States; ^4^Department of Chemical and Materials Engineering, San José State University, San José, CA, United States

**Keywords:** *pseudomonas aeruginosa*, antibiofilm, prestwick chemical library, drug repositioning, colistin, zero interaction potency, drug combinations, synergy

## Abstract

Biofilm-associated *Pseudomonas aeruginosa* infections remain a significant clinical challenge since the conventional antibiotic treatment or combination therapies are largely ineffective; and new approaches are needed. To circumvent the major challenges associated with discovery of new antimicrobials, we have screened a library of compounds that are commercially available and approved by the FDA (Prestwick Chemical Library) against *P. aeruginosa* for effective antimicrobial and anti-biofilm activity. A preliminary screen of the Prestwick Chemical Library alone did not yield any repositionable candidates, but in a screen of combinations with a fixed sub-inhibitory concentration of the antibiotic colistin we observed 10 drugs whose bacterial inhibiting activity was reproducibly enhanced, seven of which were enhanced by more than 50%. We performed checkerboard assays of these seven drugs in combination with colistin against planktonic cells, and analysis of their interactions over the complete combination matrix using the Zero Interaction Potency (ZIP) model revealed interactions that varied from highly synergistic to completely antagonistic. Of these, five combinations that showed synergism were down-selected and tested against preformed biofilms of *P. aeruginosa*. Two of the five combinations were active against preformed biofilms of both laboratory and clinical strain of *P. aeruginosa*, resulting in a 2-log reduction in culturable cells. In summary, we have identified synergistic combinations of five commercially available, FDA-approved drugs and colistin that show antimicrobial activity against planktonic *P. aeruginosa* (Clomiphene Citrate, Mitoxantrone Dihydrochloride, Methyl Benzethonium Chloride, Benzethonium Chloride, and Auranofin) as well as two combinations (Auranofin and Clomiphene Citrate) with colistin that show antibiofilm activity.

## Introduction

*Pseudomonas aeruginosa* is an opportunistic pathogen that is commonly found in soil and can survive in several niches inside hospitals. It is one of the pathogens most commonly isolated from nosocomial infections (Donlan, 2001), such as ventilator-associated pneumonia (Parker et al., 2008), catheter-associated urinary tract infections (Mittal et al., 2009), severe burn infections (Branski et al., 2009), and post-operative surgical site infections (Jombo et al., 2010). The high prevalence of *P. aeruginosa* in hospitals combined with the overuse of broad-spectrum antibiotics have led to a significant surge in drug-resistant *P. aeruginosa* (Streeter and Katouli, 2016). Although several mechanisms contribute to the ability of *P. aeruginosa* to resist antibiotics, such as drug inactivation (Majiduddin et al., 2002) and target site modification (Nakano et al., 1997), one of the most significant mechanisms is through the production of a dense exopolymeric matrix by the bacteria, known together as a biofilm. Biofilms protect the bacteria from attack by the immune system (Hoiby et al., 2011), confer resistance to antibiotics by decreasing uptake and increasing efflux (Bayer et al., 1991; Pamp et al., 2008; Hoiby et al., 2010; Alhede et al., 2011; Van Acker and Coenye, 2016), and contribute to bacterial survival and overall pathogenicity (Mittal et al., 2009). Furthermore, *P. aeruginosa* is intrinsically resistant to some common antimicrobials due to its dual-membrane nature (Lambert, 2002; Breidenstein et al., 2011), which is a characteristic of gram-negative microorganisms. Due to this intrinsic resistance to antibiotics, its ability to easily develop new resistance, its ability to create biofilms, and the recent decline in drug discovery programs (Wilkinson and Pritchard, 2015), *P. aeruginosa* infections have become an urgent worldwide health concern (Tacconelli and Magrini, 2017). Recent efforts to address this growing challenge include repositioning screens to identify commercially approved drugs with novel antimicrobial activity (Siles et al., 2013; Rangel-Vega et al., 2015; Wilkinson and Pritchard, 2015; Torres et al., 2016; Yssel et al., 2017), and combinatorial drug screens to identify combinations of traditional antibiotics and novel repositionable modulators (Delattin et al., 2014; Van den Driessche et al., 2017). In this work, we screened a library of commercially available small molecules, the Prestwick Chemical Library (PCL, a library of commercially available FDA-approved small molecules from a variety of pharmaceutical classes) in combination with sub-inhibitory concentrations of colistin (polymyxin E), against planktonic and biofilm cultures of *P. aeruginosa*. Since colistin is a well-established cationic polypeptide antimicrobial known to permeabilize the outer cell membrane of some Gram-negative bacteria (Mohamed et al., 2016), we hypothesized that exploiting this mechanism with a sub-inhibitory concentration of colistin would allow access of other drugs to their cellular targets. We also characterized the nature of the interactions between colistin and any given repositionable candidate as either synergistic, indifferent, or antagonistic based on the widely used Fractional Inhibitory Concentration Index (FIC_i_; Hall et al., 1983), and a recently-developed model for synergy scoring over the entire range of concentrations [Zero Interaction Potency (ZIP; Yadav et al., 2015)]. Finally, we have validated the activity of promising combinations on biofilms of the clinical strains *P. aeruginosa* 1244.

## Materials and methods

### Strains and culture conditions

We used the laboratory strain of *P. aeruginosa* PA01 for the preliminary screen, colistin combination screen, checkerboard assay, and biofilm screen. In addition, promising combinations that showed activity against PA01 biofilms were also tested against biofilms of the clinical strain of *P. aeruginosa* 1244 (Walker et al., 1964). Both strains were prepared as described below. Stocks were stored in cryogenic bead vials soaked in glycerol at −80°C. For every experiment, we prepared overnight cultures by transferring a single bead from the frozen stock into 20 mL of Tryptic Soy Broth (TSB) media (Becton, Dickinson & Co., Franklin Lakes, NJ) and incubating in a shaking incubator (Thermo Fisher, Waltham, MA) at 150 rpm and 37°C. For the preliminary screen, combination screen, and the checkerboard experiments, we transferred 100 μL of the overnight culture to 10 mL of fresh TSB and incubated for 3 h at 150 rpm and 37°C to allow the subculture to reach the log growth phase. Then, we transferred 1 mL aliquots of the log-phase culture into 1.5 mL micro-centrifuge tubes and centrifuged at 4,000 rpm for 15 min. After centrifugation, we washed the cells twice with sterile Phosphate-Buffered Saline (PBS, Sigma-Aldrich, St. Louis, MO) and quantified at OD_600_ in a BioPhotometer (Eppendor, Hauppaugue, NY) using PBS alone as a blank. After one last centrifugation we adjusted the density of the log-phase culture to 4 × 10^6^ cells/mL in 2 × Muller Hinton Broth (MHB) media (Becton, Dickinson & Co., Franklin Lakes, NJ). For the pre-formed, glass-disc biofilm experiments we adjusted the log-phase culture to OD_600_ of 0.05 (1.0 × 10^6^ cells/mL) in sterile PBS.

### Preliminary screen

First, we performed an initial screen of all 1,280 off-patent FDA-approved drugs in the Prestwick Chemical Library (Prestwick Chemical, Illkirch-Graffenstaden, France) at 10 μM in 96-well plates against planktonic cultures of *P. aeruginosa*. Briefly, we prepared seeding solutions at a density of 4 × 10^6^ cells/mL in 2 × MHB, and dispensed 50 μL into 96-well plates using a Hamilton Microlab STARlet robotic liquid handling system (Hamilton Robotics, Reno, NV). Next, we prepared working solutions of the Prestwick compounds in sterile milli-Q H_2_O at a concentration of 20 μM. Then we combined 50 μL of the working solutions of the compounds with the pre-seeded 96-well plates using the STARlet to create duplicate test plates with cell and compound concentrations of 2 × 10^6^ cells/mL and 10 μM, respectively. Finally, we incubated the test plates at 37°C for 24 h. After treatment, we determined cell survival using turbidometry at OD_600_ (Campbell, 2011; Sun et al., 2016) and classified compounds that reduced the turbidity of the culture by more than 50% relative to an untreated control as “hits,” which we selected for subsequent dose-response experiments.

### Colistin combination screen

In order to clearly highlight the enhanced activity of colistin combinations, we used a sub-inhibitory concentration of colistin that had no activity on its own. In preliminary studies we found that colistin had an MIC of 6.24 μg/mL against planktonic cultures of *P. aeruginosa*. Therefore, we re-screened the PCL in combination with colistin at a final concentration of 1.56 μg/mL, a concentration 4-fold lower than the MIC. Briefly, we prepared working solutions of the individual PCL drugs and colistin at final concentrations of 20 μM and 3.12 μg/mL, respectively, in sterile milli-Q H_2_O. Next, we prepared a cell-seeding solution at a density of 4 × 10^6^ cells/mL in 2 × MHB, as described in the culture conditions section. Lastly, we combined 50 μL of the drug + colistin working solutions with the cell-seeding solution, in duplicate, in new 96-well plates using the Hamilton Microlab STARlet robotic liquid handling system (STARlet, Hamilton Robotics, Reno, NV), resulting in test plates with final cell, drug, and colistin concentrations of 2 × 10^6^ cells/mL, 10 μM, and 1.56 μg/mL, respectively. Finally, we incubated the test plates at 37°C for 24 h, and determined cell survival using turbidometry at OD_600_.

### Checkerboard assays

We tested the selected drug combinations in double-dose response (checkerboard) experiments to determine the nature of the interaction. First, we prepared serial dilutions of each of the selected drugs (3.125–200 μM in sterile milli-Q H_2_O) and combined them with serial dilutions of colistin (0.78–50 μg/mL in sterile milli-Q H_2_O). Next, we prepared a cell-seeding solution at a density of 4 × 10^6^ cells/mL in 2 × MHB, as described in the culture conditions section. Lastly, we combined 50 μL of the dilution combination plates with the cell-seeding solution in new 96-well plates, in duplicate, using the STARlet, resulting in test plates with final cell, drug, and colistin concentrations of 2 × 10^6^ cells/mL, 0.78 μM-50 μM, and 0.195 μg/mL-12.5 μg/mL, respectively (**Figure 2**). For controls, we seeded a column and row of drug or colistin alone, as well as untreated and dead cell control wells accordingly. Finally, we incubated the test plates at 37°C for 24 h and determined cell survival using turbidometry at OD_600_.

### Qualification of interactions

We determined the nature of the interaction between the two drugs based on the combined and individual antimicrobial activities based on an established method of synergy (Hall et al., 1983). Briefly, we calculated individual MICs when possible, and compared with the concentration of each drug in isoeffective (having the same killing effect as the individual MICs) combinations. From this data, we calculated the FIC_i_ for select combinations using the Lowe model of synergy:

$$\begin{array}{l} FIC_{i}=\left(\frac{{C_{A}}^{Combo}}{{MIC_{A}}^{Alone}}\right)+\left(\frac{{C_{B}}^{Combo}}{{MIC_{B}}^{Alone}}\right). \end{array}$$

We then labeled the interaction between colistin and the drug in each combination as synergistic if the FIC_i_ was equal to or below 0.5, indifferent if it was between 0.5 and 4, or antagonistic if it was over 4. Next, we evaluated the entire combination matrix for synergy or antagonism using the ZIP model (Yadav et al., 2015). For this analysis we used the R package “synergyfinder” (https://bioconductor.org/packages/release/bioc/html/synergyfinder.html). Lastly, we selected synergistic and nearly-synergistic combinations for further testing against pre-formed biofilms.

### Formation of biofilms on glass discs

We used a model of biofilm formation that has been previously described (Miller et al., 2016). First, we adjusted log-phase cultures of *P. aeruginosa* to an OD_600_ of 0.05 in sterile PBS (seeding solution). Then, we submerged sterile glass discs (9 mm × 1.75 mm; Ace Glass Inc., Vineland, NJ) in 1 ml of the seeding solution in a 48-well plate for 2–3 h to allow for cell attachment to the glass surface. After cell attachment, we transferred the glass discs to 1 mL of BHI++ media (BHI supplemented with 2% NaCl and 1% Glucose) in a new 48-well plate and incubated at 37°C for 24 h to allow for biofilm formation.

### Confocal laser scanning microscopy of pre-formed biofilms

The successful formation of biofilms on the glass surface was confirmed by Confocal Laser Scanning Microscopy (CLSM). A Filmtracer Live/Dead Biofilm Viability Kit (Cat. number L10316, Thermo Fisher, Waltham, MA) was used to stain cells within the extracellular matrix. Glass discs were submerged in a 10 μM SYTO9 staining solution for 30 min, followed by a 60 μM Propidium Iodide (PI) staining solution for 30 min. After staining, the glass discs were rinsed in PBS and imaged in a Zeiss LSM 510 Upright Confocal Microscope (Carl Zeiss, Thornwood, NY) at 40X magnification (Zeiss Achroplan water immersion lens 40X/0.8W). Images were analyzed with the Fiji image processing package for ImageJ.

### Activity of synergistic combinations against pre-formed *P. aeruginosa* biofilms

We tested synergistic and nearly-synergistic combinations, as determined above, against pre-formed biofilms of *P. aeruginosa* to determine their anti-biofilm potential.

After biofilm formation, we rinsed the glass discs three times in PBS to remove any unattached cells and transferred them to 48-well plates containing BHI++ media with the appropriate drugs/drug combinations and incubated at 37°C for 24 h to allow for drug action. After treatment, we rinsed the glass discs in PBS and transferred to 1 mL PBS in a 15 mL conical tube, and sonicated in a Microson XL ultrasonic cell disruptor (Qsonica, LLC, Newtown, CT) for 2 min to detach the biofilm from the glass surface. Next, we prepared five 10-fold serial dilutions from each biofilm sample and plated in triplicates on blood agar plates. Finally, we incubated the plates at 37°C and after 24 h counted the colonies on the plates using an automated colony counter (ProtoCOL-Synbiosis, Microbiology International, Frederick, MD). The combinations that showed activity against PA01 biofilms were tested as above against the clinical *P. aeruginosa* strain 1244.

## Results

### Preliminary screen

**Figure 1:**
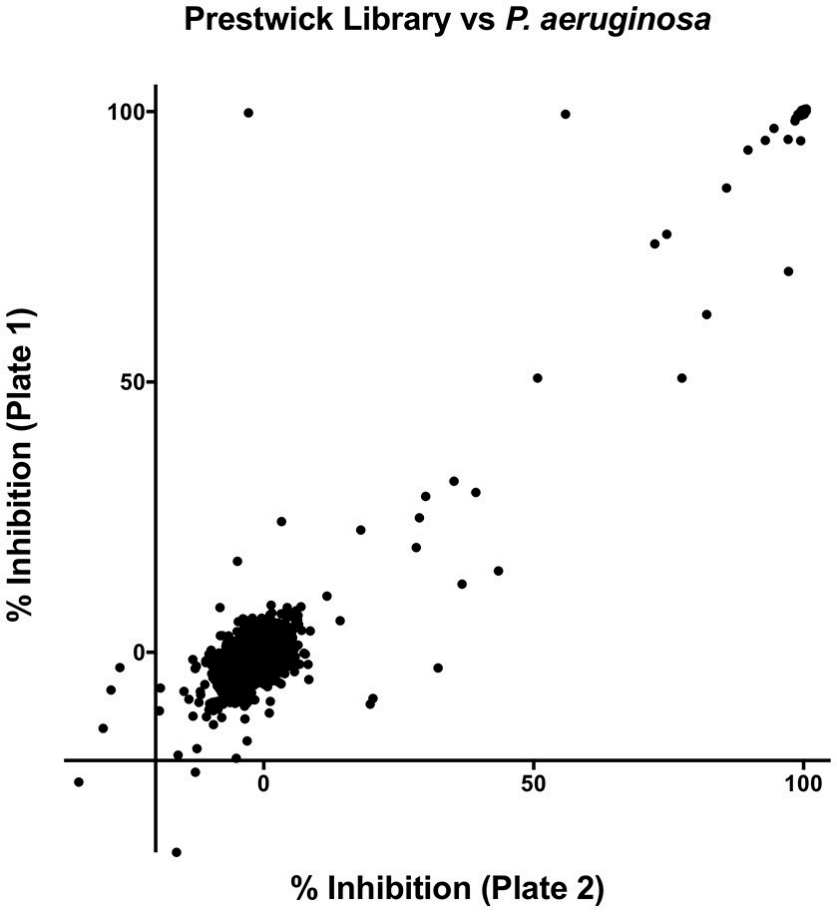
Results of the primary screen, 34 drugs inhibited growth of *P. aeruginosa* by 50% or more.

The preliminary screen of the PCL compounds against planktonic cultures of *P. aeruginosa* yielded 34 “hits” that reduced cell survival below 50% (Figure 1). All 34 hits were antimicrobials of various classes, including β-lactams, fluoroquinolones, and aminoglycosides, and 26 of the 34 compounds inhibited cell growth by more than 90% (Table 1). However, none of the other compounds displayed any antimicrobial activity.

**Table 1:** Drugs with activity against planktonic *P. aeruginosa*.

**Drug**	**Class**	**% Inhibition at 10 μM**
Cefotaxime sodium salt	Cephalosporin	50.7
Aztreonam	Monolactam	98.7
Cefoperazone dihydrate	Cephalosporin	98.4
Colistin sulfate	Polymyxin	99.5
Dirithromycin	Macrolide	73.9
Ceftazidime pentahydrate	Cephalosporin	83.8
Piperacillin sodium salt	Penicillin	77.3
Azlocillin sodium salt	Penicillin	99.4
Cefsulodin sodium salt	Cephalosporin	72.3
Cefepime HCl	Cephalosporin	99.8
Azithromycin	Macrolide	76.1
Cefpiramide	Cephalosporin	99.4
Ciprofloxacin HCl	Fluoroquinolone	99.7
Dihydrostreptomycin sulfate	Aminoglycoside	99.8
Gentamicin sulfate	Aminoglycoside	99.9
Norfloxacin	Fluoroquinolone	97.1
Lomefloxacin HCl	Fluoroquinolone	64.1
Streptomycin sulfate	Aminoglycoside	99.7
Amikacin hydrate	Aminoglycoside	99.9
Tosufloxacin HCl	Fluoroquinolone	99.9
Tobramycin	Aminoglycoside	99.9
Sisomycin sulfate	Aminoglycoside	99.9
Merbromin	Organomercuric	99.8
Clinafloxacin	Fluoroquinolone	99.9
Apramycin	Aminoglycoside	96.1
Sarafloxacin	Fluoroquinolone	99.9
Rifabutin	Ansamycin	85.8
Gatifloxacin	Fluoroquinolone	99.9
Moxifloxacin	Fluoroquinolone	93.8
Fleroxacin	Fluoroquinolone	99.3
Enoxacin	Fluoroquinolone	99.9
Sparfloxacin	Fluoroquinolone	99.9
Rifaximin	Ansamycin	91.3
Besifloxacin HCl	Fluoroquinolone	99.9

### Colistin combination screen

Next, we re-screened the compounds from the PCL in the presence of sub-inhibitory concentration of colistin (1.56 μg/mL, 4-fold lower than MIC). From this re-screening we identified seven compounds that were “enhanced” by a sub-inhibitory concentration of colistin, reducing the survival of *P. aeruginosa* cultures by more than 50% when compared to the drug alone (Table 2). The seven drugs were of varying classes, including antineoplastics, fertility, analgesics, and antibacterials, which previously had no activity against *P. aeruginosa* on their own.

**Table 2:** Percent survival of planktonic *P. aeruginosa* after treatment with drug alone and in combination with 1.56 μg/mL Colistin.

**Plate/well**	**Drug name**	**Class**	**Alone**	**Combination**	**Structure**
5/G6	Mitoxantrone DHCl	Antineoplastic	107.2	50.43	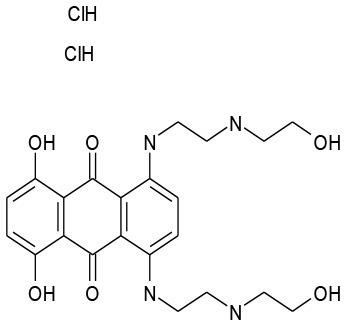
5/H8	Clomiphene Citrate	Endocrinology	103.6	37.6	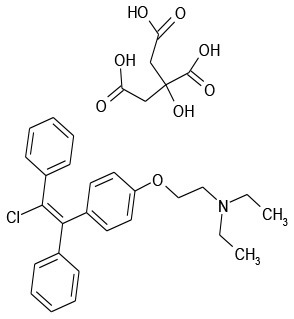
7/E3	Thiostrepton	Antibacterial	94.9	41.9	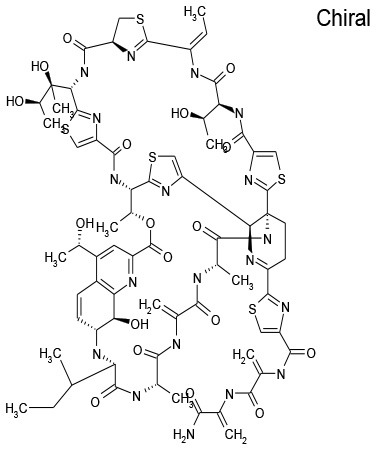
9/G6	Methyl Benzethonium Cl	Antibacterial	107.8	0.13	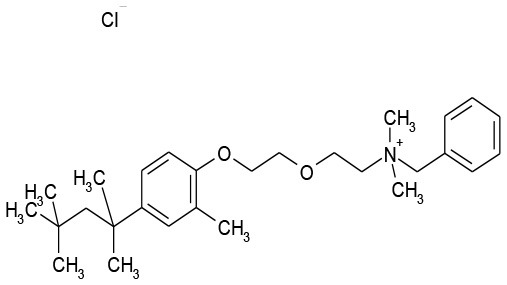
9/G9	Benzethonium Cl	Antibacterial	103.6	33.8	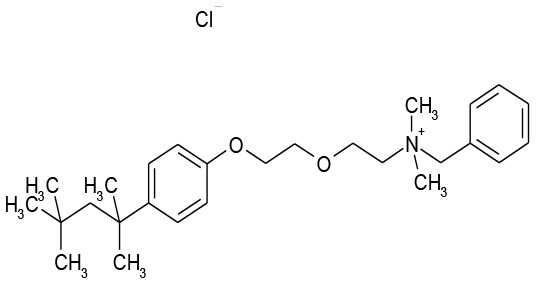
11/B2	Auranofin	Analgesic	126.5	7.5	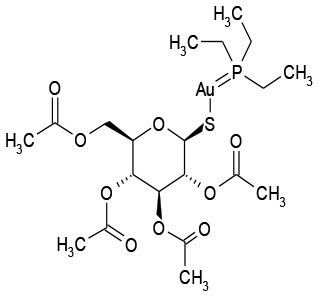
12/E6	Thonzonium Br	Antiseptic	117.4	0.3	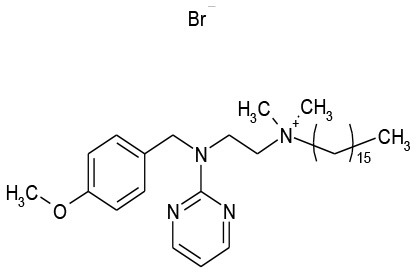

### Checkerboard assays

**Figure 2:**
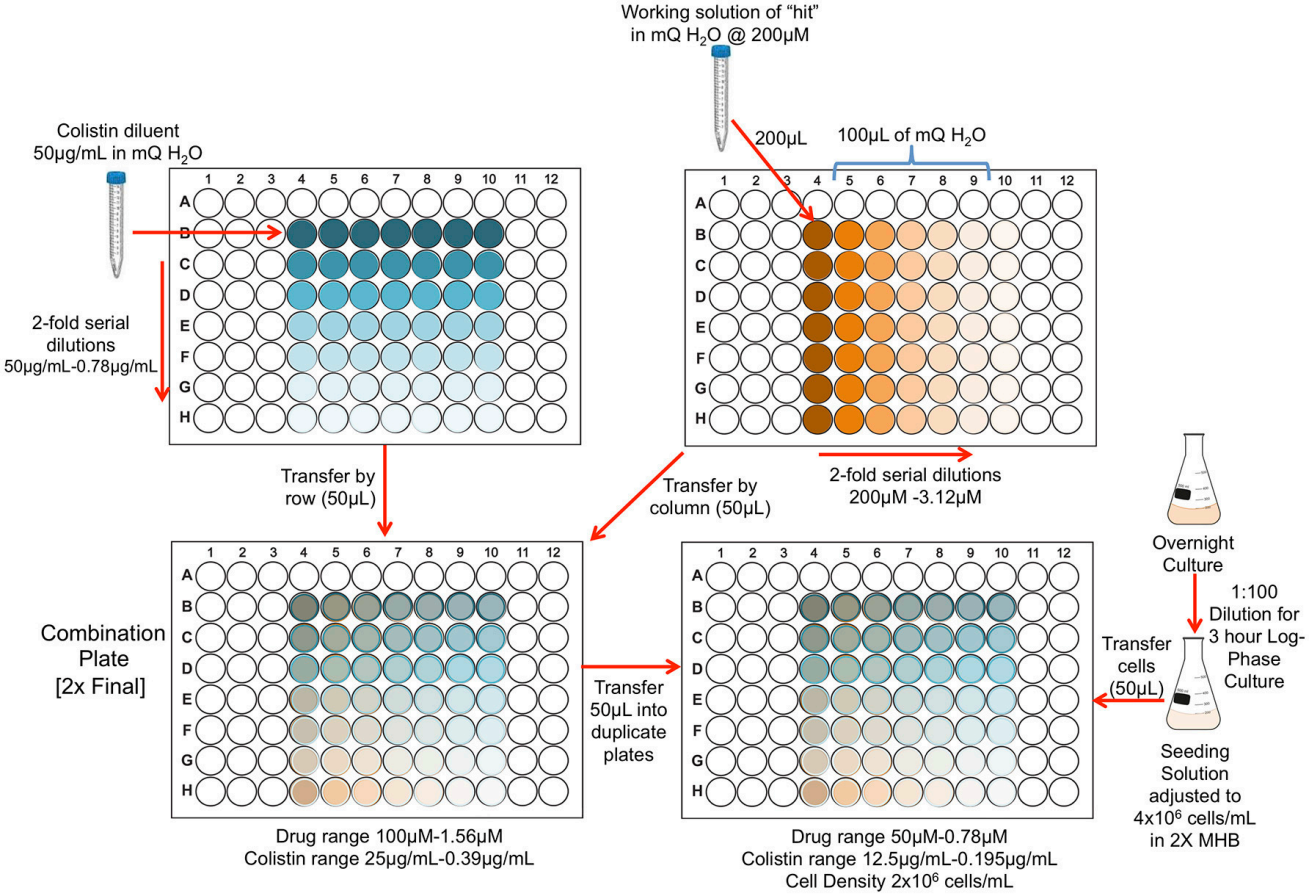
Schematic of checkerboard assay.

**Figure 3:**
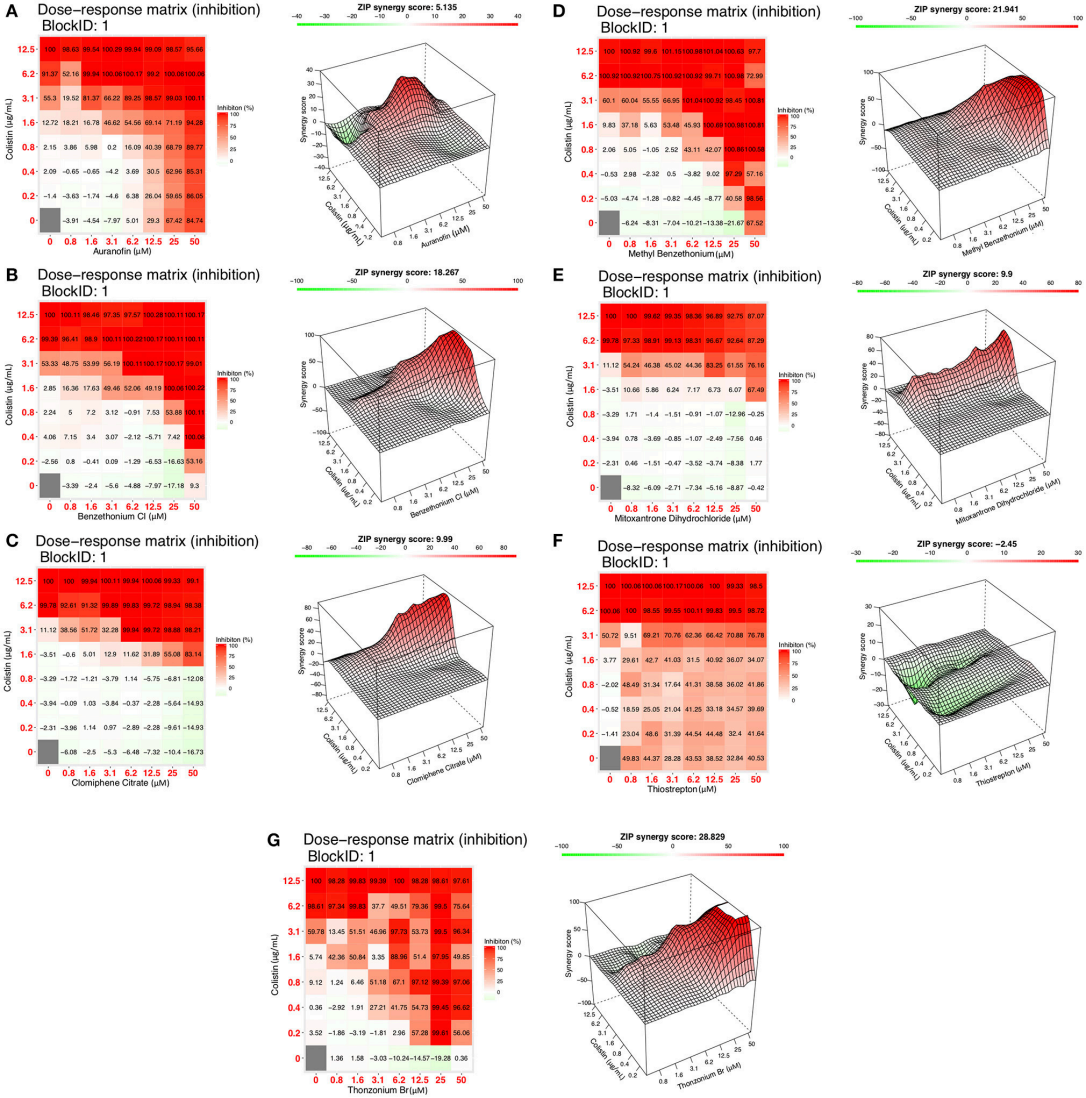
Dose response matrix and ZIP synergy score surface plots of **(A)** Auranofin, **(B)** Benzethonium Cl, **(C)** Clomiphene Citrate. Dose response matrix and ZIP synergy score surface plots of **(D)** Methyl Benzethonium, **(E)** Mitoxantrone DHCl, **(F)** Thiostrepton. Dose response matrix and ZIP synergy score surface plots of **(G)** Thonzonium Br (μM).

The seven “hits,” repositionable drugs, referred to as simply “candidates,” were then tested in double dose-response (checkerboard) assays to determine their individual and combination potencies (Figure 2). The MICs of the individual drugs could not be determined for most of the seven candidates, as these drugs were ineffective against *P. aeruginosa* even at a concentration as high as 50 μM. Six candidates, clomiphene citrate, mitoxantrone dihydrochloride, auranofin, benzethonium chloride, thonzonium bromide, methyl benzethonium chloride, showed various degrees of increased antipseudomonal activity when combined with colistin when compared to drug or colistin alone (Figure 3). Thiostrepton, however, showed no increase or decrease of activity at any colistin concentration.

### Qualification of interactions of candidates with colistin

Next, we determined the nature of the interaction between colistin and each of the repositionable drugs. Currently, there is no standardized methodology by which drug interactions are characterized, although a few useful approaches exist. For this study, we followed the Fractional Inhibitory Concentration method described by Hall et al. (1983), which uses a FIC_i_ to determine whether the interaction between two drugs is either antagonistic, indifferent, or synergistic. The survival data from checkerboard assays was used to determine the FIC_i_ of each combination, using the Lowe model described in the Methods section. However, the Minimum Inhibitory Concentration (MIC) of each individual drug must be determined in order to calculate the FIC_i_. For colistin, the MIC has been described against several strains of *P. aeruginosa* (Moskowitz et al., 2010), which match up well with our findings (6.2 μg/mL). In dose response studies (data not shown), we found that Benzethonium Cl had an MIC of 50 μM. However, in the case of the remaining six candidates in question (Table 2, Figure 3) the MICs were above the highest concentration tested (>50 μM). In order to calculate the FIC_i_ of these, we assigned the highest concentration tested (50 μM) as the MIC in Equation 1. Because of this limitation, the following are only partial and reasonable characterizations of the interaction between any two drugs. In fact, this analysis is conservative since a higher MIC value of any of the individual candidates would decrease the corresponding FIC_i_, indicating that the combination is “more synergistic.” Based on the criteria set forth by the Journal of Antimicrobial Chemotherapy (Odds, 2003) and Antimicrobial Agents Chemotherapy (2017) regarding the interpretation of FIC_i_ values, only three of the seven drugs—auranofin, thonzonium bromide, and methyl benzethonium chloride—had a synergistic interaction with colistin, whereas the remaining four—clomiphene citrate, mitoxantrone dihydrochloride, methyl benzethonium chloride, and thiostrepton—had an indifferent interaction (Table 3).

**Table 3:** Selected Drug/Colistin combinations, FIC indexes, and corresponding classification (Colistin/Drug).

**Drug**	**Class**	**FIC_i_**	**Interaction**	**Concentration at FIC_i_**	**Most synergistic combination (ZIP)**
Clomiphene citrate	Endocrinology	0.63	Indifferent	3.12 μg/mL/6.25 μM	–
Mitoxantrone DHCl	Antineoplastic	0.75	Indifferent	3.12 μg/mL/12.5 μM	–
Thiostrepton	Antibacterial	1.01	Indifferent	6.24 μg/mL/0.5 μM	–
Methyl benzethonium	Antibacterial	0.50	Synergistic	1.56 μg/mL/12.5 μM	0.4 μg/mL/50 μM
Benzethonium Cl	Antibacterial	0.63	Indifferent	3.12 μg/mL/6.25 μM	–
Auranofin	Analgesic	0.52	Synergistic	6.24 μg/mL/1.56 μM	3.12 μg/mL/6.24 μM
Thonzonium Br	Antiseptic	0.53	Synergistic	6.24 μg/mL/1.56 μM	0.4 μg/mL/50 μM

Since the FIC index estimates the drug interactions only at MIC levels (or any other predetermined level), we used ZIP analysis to understand the interactions over the entire range of concentrations. This new method, developed by Yadav et al. (2015), allows us to simultaneously score all concentration combinations (as opposed to just one at a time as in the FIC_i_ method) based on the deviation of the IC_50_ (concentration that gives half-maximal response) and sigmoidicity parameters of the logistic model used to fit the individual drug curves. Surface response plots of these synergy scores allow us to look at the overall landscape of synergy giving us a more complete understanding of the interaction between the two drugs. This method is based on the assumption that drugs that do not interact will simply affect the baseline of each other's response curve, but not the shape of the curve (sigmoidicity) or concentration necessary to achieve a half-maximal response (IC_50_). Therefore, any deviation in these values observed when combining with another drug would indicate synergism or antagonism. The results are shown in Figure 3 for the seven candidates, alongside the FIC_i_ plots. Surface plots (panels on the right) of the model-specific statistic (which is the average of the deviations of shape and potency parameters before and after combination with another drug and represents the synergy score, also known as the delta value) show regions of synergy in six of the seven drug-pair matrices (auranofin, thonzonium bromide, methyl benzethonium chloride, clomiphene citrate, mitoxantrone dihydrochloride, benzethonium chloride, (right panels of Figures 3A–E,G); with average ZIP synergy scores ranging from 5.1 (moderate synergism) to 28.8 (high synergism). On the other hand, regions of antagonism are present in the thiostrepton matrix (Figure 3F).

### Activity of synergistic combinations against pre-formed *P. aeruginosa* biofilms

**Figure 4:**
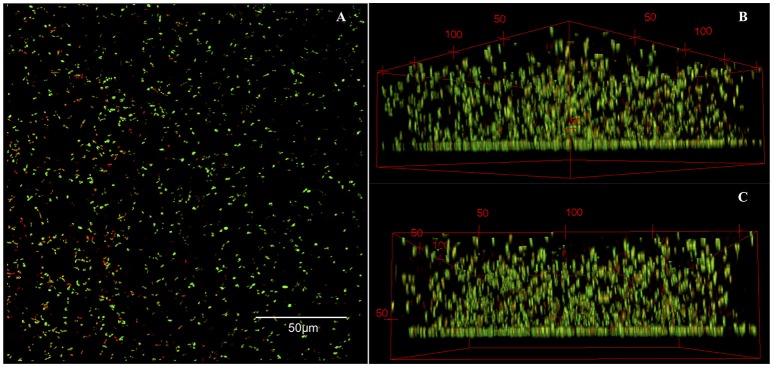
CLSM images of a biofilm formed on a glass surface. **(A)** An overhead composite shows the presence of live and dead cells, while the 3D **(B)** corner and **(C)** side views illustrate the overall structure of the biofilm, including a surface-attached layer. **(B)** and **(C)**, the numbers indicate scale in μM.

To investigate the effect of synergistic combinations of the repositionable drug candidates and colistin against *P. aeruginosa* biofilms, we formed biofilms on glass disc. The formation of biofilms on the glass disc surface was confirmed by CLSM. Figure 4 shows an overhead composite projection (Figure 4A), as well as 3D corner (Figure 4B), and side views (Figure 4C) of a representative biofilm. A surface-attached layer at least 70–100 μm thick is clearly visible, accompanied by live and dead cell clusters suspended in the extracellular matrix. These features, a surface-attached layer and 3-dimensional structure, are defining characteristics of biofilms, and serve as confirmation that we successfully formed *P. aeruginosa* biofilms on the glass disc surface.

**Figure 5:**
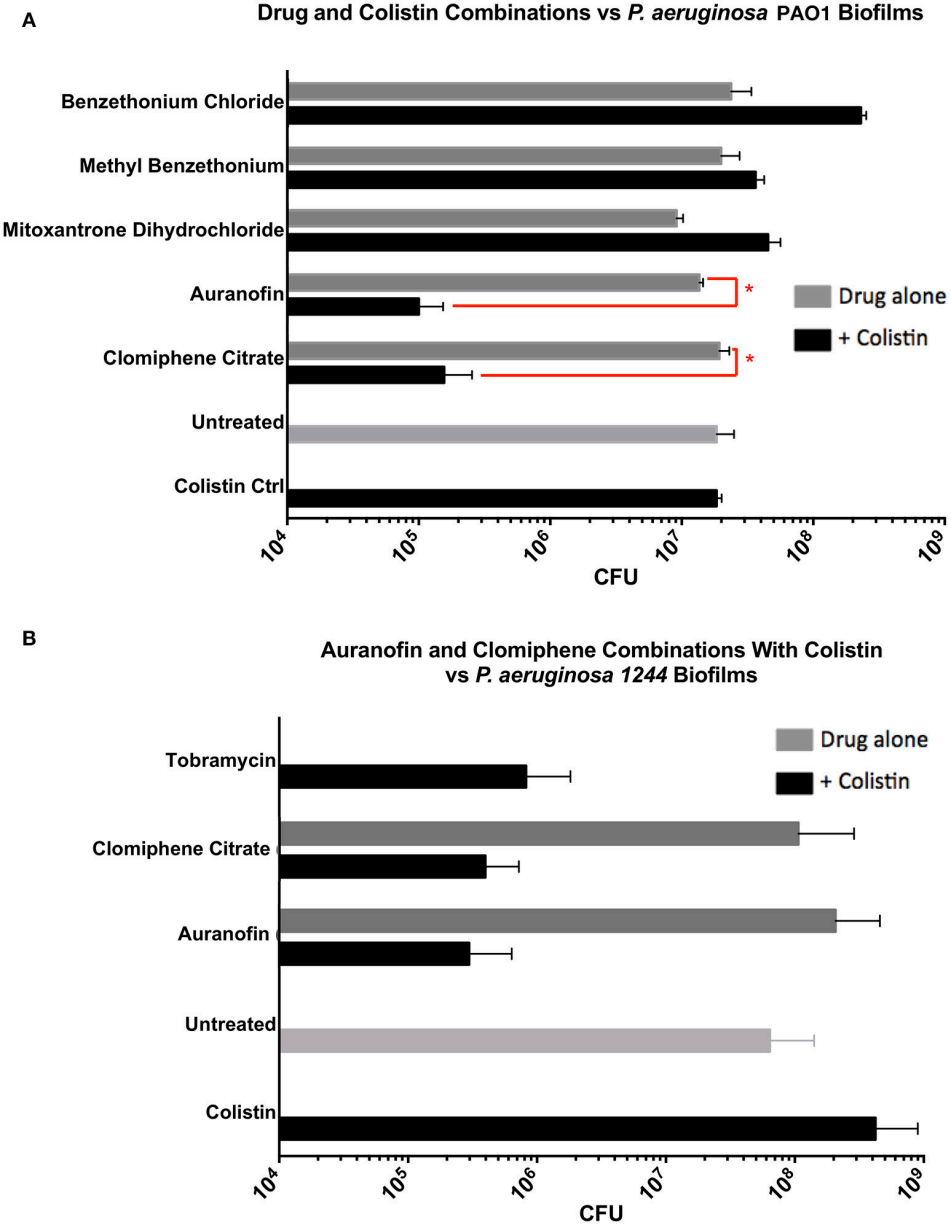
**(A)** Pre-formed biofilms of *P. aeruginosa* (PA01) were treated with selected Colistin combinations. Only two combinations, Auranofin/Colistin (1.56 μM/6.24 μg/mL) and Clomiphene Citrate/Colistin (6.25 μM/3.12 μg/mL), showed activity against biofilms. **(B)** Pre-formed biofilms of *P. aeruginosa* 1244 were treated with Auranofin/Colistin (1.56 μM/6.24 μg/mL) and Clomiphene Citrate/Colistin (6.25 μM/3.12 μg/mL). As with PA01, there was a 2-log reduction in 1244 cell survival. ^*^P < 0.05, *n* = 3.

Lastly, we tested two synergistic combinations and three nearly-synergistic combinations (as determined by the FIC_i_ method: auranofin, clomiphene citrate, mitoxantrone dihydrochloride, benzethonium chloride, and methyl benzethonium chloride) against pre-formed biofilms. As can be seen from Figure 5A and as expected, the drugs, by themselves, did not have any effect on the biofilms. Combinations of colistin and methyl benzethonium chloride, and mitoxantrone dihydrochloride, had no appreciable effect on *P. aeruginosa* biofilm survival; on the other hand, combinations of colistin and clomiphene citrate, as well as colistin and Auranofin, showed significant killing corresponding to a 2-log reduction in culturable cells compared to individual drugs and untreated controls (Figure 5A). To confirm the results obtained with laboratory strain on a clinical strain, these two combinations were further tested against pre-formed biofilms of *P. aeruginosa* clinical strain 1244. Colistin and clomiphene citrate, and colistin and auranofin had activity against biofilms of the 1244 strain corresponding to a 2-log reduction in cell survival (Figure 5B). These results allowed us to identify the two drug combinations that are effective against recalcitrant *P. aeruginosa* biofilms.

## Discussion

The goal of this work was to find repositionable candidates with no previously-reported antimicrobial activity against *P. aeruginosa* biofilms, which are notorious for their heightened drug resistance. Since all of the 34 hits from the preliminary screen of the PCL were antimicrobials or antiseptics with well-established activity against *P. aeruginosa* (Table 1), we re-screened the entire PCL combined with a sub-inhibitory concentration of colistin. From this combination re-screening, we identified seven drugs (repositionable candidates) of different classes with activity “enhanced” by colistin, reducing planktonic *P. aeruginosa* survival by more than 50% when compared to the drug or colistin alone: anti-neoplastics (mitoxantrone dihydrochloride), fertility (clomiphene citrate), antirheumatics (auranofin), and antibacterials with no previously described activity against *P. aeruginosa* (thiostrepton, methyl benzethonium chloride, and thonzonium bromide), and six of the seven combinations show regions of moderate to high synergism. To our knowledge, this is the first report describing the antimicrobial activity of these candidates, either alone or in combination, against *P. aeruginosa* biofilms, and for some candidates, against any known common pathogens. While the mechanisms of action are not yet known, we suggest the following as the putative mechanisms based on published reports in other systems. Furthermore, the widely different chemical structures of the candidates may warrant a variety of approaches to unravel these mechanisms.

There are limited reports of the antimicrobial activity of mitoxantrone, an antineoplastic agent, mostly concerning its activity in combination with antimicrobials for the treatment of infections arising during chemotherapy. Falchi et al. (1989) found that mitoxantrone did not interact with ceftazidime. Other reports found indifference between mitoxantrone and some other antimicrobials, such as ceftriaxone and piperacillin (Gieringer et al., 1986). In our studies, we found that Mitoxantrone has no activity alone against *P. aeruginosa*, but observed a significant increase in activity when combined with colistin (Figure 3E).

Clomiphene citrate, a fertility drug, has been shown to have antimicrobial activity against Gram-positives *Staphylococcus aureus, Bacillus subtilis* (Farha et al., 2015), and *Enterococcus faecium* (Jacobs et al., 2013). Furthermore, Tamoxifen, an analog of Clomiphene has been shown to have antifungal activity against *Saccharomyces cerevisiae* (Wiseman et al., 1989), *Cryptococcus neoformans*, and *Candida albicans* (Dolan et al., 2009). It has been determined that clomiphene citrate targets the cytoplasmic enzyme undecaprenyl diphosphate synthase (UppS), which synthesizes the lipid carrier protein undecaprenyl phosphate (Und-P) responsible for transporting a major substrate in wall-techoic acid (WTA) synthesis (Farha et al., 2015). Inhibition of synthesis of WTAs, a major component of the Gram-positive cell wall, is the source of the antimicrobial activity of clomiphene citrate. However, there are limited reports on the activity of clomiphene citrate against Gram-negative pathogens. Consistent with a previous report on the lack of any significant activity of clomiphene citrate against *P. aeruginosa* and *Escherichia coli*, we observed no activity against *P. aeruginosa* (Prasad et al., 2011). However, we observed a significant increase in activity when combined with colistin (Figure 3C). Although WTAs are not present in Gram-negatives, undecaprennyl phosphate carrier lipids are still involved in the synthesis and transport of lipopolysaccharide precursors in *E. coli*, and are also presumed so in *P. aeruginosa* (King et al., 2009). The synthesis of these undecaprennyl phosphate carrier lipids may be the target of clomiphene citrate as in Gram-positives, and we believe colistin may provide the necessary access to this process.

Auranofin, an antirheumatic agent, has received significant attention in recent years due to its wide-ranging activity as an antimicrobial agent against Gram-positive bacteria (Cassetta et al., 2014; Aguinagalde et al., 2015; Harbut et al., 2015; Thangamani et al., 2016) and fungal pathogens (Fuchs et al., 2016). Auranofin, however, has limited activity against some gram-negative bacteria, including *P. aeruginosa* (Harbut et al., 2015). Thangamani et al propose that this lack of activity is due to the inability of auranofin to penetrate the outer cell membrane of Gram-negative bacteria (Thangamani et al., 2016). Their conclusion is supported by our finding that a combination of colistin, with its permeabilizing action, and auranofin has strong anti-pseudomonal activity compared to either drug alone (Figure 3A). At the time of publication of this work, we became aware of as yet unpublished (preprint) work by Tan et. al., demonstrating that in combination with colistin, auranofin has significant killing activity against pre-formed biofilms of *P. aeruginosa* PA01 in flow cell and mouse models through the inhibition of the global regulator (transcription factor) of virulence factor expression, Vfr, which is central to the expression of the quorum sensing mechanism, secretion systems, and formation of pili necessary for twitching motility (Tan et al., 2017). Their observations agree with our findings and shed light on the mechanism by which the auranofin-colistin combination disrupts, and eventually kills, pre-formed biofilms.

Benzethonium chloride and methyl benzethonium chloride are quaternary ammonium salts whose antimicrobial activity has been studied since the 1930s. There are even early reports of synergistic antimicrobial and antifungal activity with acylated peptides (Liebert, 1985). Of note, colistin is a polymyxin peptide with a fatty acyl chain. Furthermore, methyl benzethonium chloride has recently been banned by the FDA for use in antibacterial soaps, and a similar ruling regarding benzethonium chloride has been delayed until 2019 (Food and Drug Administration, 2016). Despite the wide-ranging activity of these compounds, they have limited activity against *P. aeruginosa* (Joslyn et al., 1943) due to their inability to penetrate the outer membrane (Jennings et al., 2015). Like other quaternary ammonium cation antimicrobials, benzethonium chloride, and methyl benzethonium chloride target the cell membrane causing leakage of intracellular components and cell death. In our studies, we observed a significant increase in activity of these compounds against *P. aeruginosa* when combined with colistin (Figures 3B,D).

Of interest to combination therapy, ZIP analyses provide important insights into drug synergism than the conventional FIC_i_ calculations as exemplified by the effect of colistin with clomiphene citrate or auranofin on *P. aeruginosa* viability. Clomiphene citrate-colistin combination had an FIC_i_ of 0.63 and was classified as “indifferent” under ASM's criteria. This “indifferent combination with antibiofilm properties” highlights an important shortcoming of the FIC_i_ method. The subjective nature of FIC_i_ interpretation could easily result in combinations that are misclassified, especially when dealing with FIC_i_ values near the arbitrary thresholds. We can see from Figure 3C that the combination that was tested (3.12 μg/mL colistin + 6.25 μM clomiphene citrate) was highly effective (99% planktonic inhibition) despite not having a low enough fraction to be classified as synergistic. The remaining candidate combinations did not show any antibiofilm activity. On the other hand, the concentration combinations that result in the lowest fractions per the FIC_i_ method (therefore more synergistic) do not necessarily have the highest ZIP synergy scores. For instance, a concentration combination of 6.24 μg/mL colistin and 1.56 μM auranofin results in 99.9% inhibition and an FIC_i_ of 0.52 (Figure 3A, Table 3), which is considered strongly synergistic. However, ZIP synergy score representing the most synergistic combinations in the matrix correspond to a much higher Auranofin concentration (3.1 μg/mL colistin and 6.2 μM Auranofin). In other words, the concentration combination that is most synergistic as per the FIC_i_ is not in fact the most synergistic concentration combination in the matrix.

In summary, we have screened a library of commercially available, FDA approved drugs (Prestwick Chemical Library) in combination with colistin, a drug known to permeabilize the outer membrane of Gram-negative bacteria, and found two synergistic and three near-synergistic combinations that have strong activity against planktonic *P. aeruginosa*. Of these five combinations only two, auranofin + colistin and clomiphene citrate + colistin, showed a 2-log reduction of pre-formed biofilms of both laboratory strain PA01 and clinical strain 1244 of *P. aeruginosa*. These two combinations should be further validated for effectiveness in an animal model of biofilm infection as necessary for in establishing pre-clinical relevance of any new therapy, especially those seeking to expand the indications of existing pharmaceuticals.

## Author contributions

NT, KL, AR, and JL-R conceived and designed the study. NT, DM-J, and JA acquired the data. NT and AS analyzed the data. NT drafted the manuscript with revisions and final approval by KL, AR, and JL-R.

### Conflict of interest statement

The authors declare that the research was conducted in the absence of any commercial or financial relationships that could be construed as a potential conflict of interest.
